# Isolation of cellulolytic bacteria from the intestine of *Diatraea saccharalis* larvae and evaluation of their capacity to degrade sugarcane biomass

**DOI:** 10.1186/s13568-015-0101-z

**Published:** 2015-02-25

**Authors:** Karina I Dantur, Ramón Enrique, Björn Welin, Atilio P Castagnaro

**Affiliations:** Estación Experimental Agroindustrial Obispo Colombres (EEAOC) - Consejo Nacional de Investigaciones Científicas y Técnicas (CONICET), Instituto de Tecnología Agroindustrial del Noroeste Argentino (ITANOA), 3150 William Cross Av., Las Talitas, PC T4101XAC Tucumán Argentina

**Keywords:** Bacterial symbionts, Cellulase, Endo-glucanase activity, Gut microbiota, Lignocellulose, Plant-insect-bacterial interaction, Sugarcane

## Abstract

As a strategy to find efficient lignocellulose degrading enzymes/microorganisms for sugarcane biomass pretreatment purposes, 118 culturable bacterial strains were isolated from intestines of sugarcane-fed larvae of the moth *Diatraea saccharalis.* All strains were tested for cellulolytic activity using soluble carboxymethyl cellulose (CMC) degrading assays or by growing bacteria on sugarcane biomass as sole carbon sources. Out of the 118 strains isolated thirty eight were found to possess cellulose degrading activity and phylogenetic studies of the *16S rDNA* sequence revealed that all cellulolytic strains belonged to the phyla γ-Proteobacteria, Actinobacteria and Firmicutes. Within the three phyla, species belonging to five different genera were identified (*Klebsiella*, *Stenotrophomonas, Microbacterium, Bacillus* and *Enterococcus*). Bacterial growth on sugarcane biomass as well as extracellular endo-glucanase activity induced on soluble cellulose was found to be highest in species belonging to genera *Bacillus* and *Klebsiella*. Good cellulolytic activity correlated with high extracellular protein concentrations. In addition, scanning microscopy studies revealed attachment of cellulolytic strains to different sugarcane substrates. The results of this study indicate the possibility to find efficient cellulose degrading enzymes and microorganisms from intestines of insect larvae feeding on sugarcane and their possible application in industrial processing of sugarcane biomass such as second generation biofuel production.

## Introduction

The imminent need to replace fossil-based transport fuels with more environment-friendly renewable alternatives, has sparked an increasing interest in finding abundant and cheap resources for biofuel production. One of the most interesting and promising alternatives, in the short- and medium-term perspective, is the second generation bioethanol (B2G) produced from lignocellulosic byproducts of agricultural, forestry and industrial activities or from urban waste residues. Natural occurring lignocellulose material, especially in the form of plant cell wall material, is a renewable, abundant and relatively cheap mixture of organic materials, principally containing polysaccharides (~75% dry weight) and lignin (~25% dry weight). The carbohydrates consist mainly of fibers of cellulose (glucose units) and hemicellulose (composed of various 5- and 6-carbon sugars), which give strength to plant structures. Lignin on the other hand, is formed by a non-carbohydrate complex structure built from phenylpropanoid units. This phenolic polymer sticks to the polysaccharide components, strengthening the whole structure which renders it extremely resistant to biological degradation (Cheng and Wang 2013).

Although much effort and resources have been directed to develop industrial scale bioethanol production from lignocellulose, there is still no economically viable industrial production system available for any type of biomass (Limayem and Ricke 2012; Morone and Pandey 2014). One of the main reasons for the high production cost of B2G is the recalcitrance of the lignocellulose to enzymatic hydrolysis, which requires a pre-treatment step before an efficient enzyme-based degradation of the complex polysaccharides (cellulose or hemicellulose) into its fermentable monosaccharide components can be carried out (Canilha et al. 2012; Cardoso et al. 2012). Currently available pretreatment methods are biological, chemical (cellulose solvents, acids, or bases), or physical (mechanical size reduction, comminution, steam explosion, vibratory ball milling, compression milling, and hydrothermolysis) (Taherzadeh and Karimi 2007; Sierra et al. 2011). Even though applying a pretreatment of the plant biomass large amounts of enzymes are still needed to obtain a rapid and efficient cellulose and hemicelluloses degradation, and therefore new and more efficient enzyme cocktails are needed in order to generate a more economic degradation process rendering a cheaper total bioethanol production.

Natural lignocellulose degradation, an essential part of the carbon cycle, is carried out by highly specialized wood-degrading microorganisms (fungi and bacteria) and symbiotic microbes found in the intestines of many plant feeding animals (Watanabe and Tokuda, 2010; Cardoso et al. 2012; Gupta et al. 2012; He et al. 2013). Hydrolysis of cellulose is a multi-enzymatic process involving at least three different types of enzymatic activities in order to liberate the smallest basic unit, a glucose molecule. First, endo-β-glucanases (E.C. 3.2.1.4) cleave the cellulose backbone at internal amorphous sites, reducing the chain length while creating numerous ends where exo-β-glucanases (E.C.3.2.1.91) attack to release short-chain glucose oligomers (cellodextrins and cellobiose). There are two forms of exo-glucanases, the first attacking the reducing end of a cellulose chain while the second attacks the non-reducing ends. Finally, the short glucose chains released after exo-glucanase attacks are hydrolyzed to single glucose units by a β-glucosidase (E.C. 3.2.1.21) (Singh and Hayashi 1995; Teeri 1997).

It is thought that the highly effective plant biomass degrading capacity found in intestines of many insect herbivores constitutes one of the most efficient naturally occurring bioreactors and could provide an important and interesting biotechnology source of microorganisms and enzymes for cellulose degradation (Sun and Scharf 2010). The sugarcane stalks borer *Diatraea saccharalis* is the major sugarcane pest in Argentina causing considerable damage to infested plants, which facilitates secondary fungal and/or bacterial infections, resulting in important economic losses to producers. Due to the high and rapid sugarcane feeding capacity of larvae of this species, we hypothesized that larvae only fed on sugarcane, could have developed a consortium community of symbiotic-bacteria possessing an enzyme arsenal which efficiently degrade sugarcane plant tissue. Supporting this theory are recently published studies where a comparison among gut microbiota from Coleoptera, Lepidoptera and Orthoptera species, show correlation between the cellulolytic enzyme activities in the insect gut with the lignocellulosic biomass composition in the food consumed by the insect (Shi et al. 2011; Cardoso et al. 2012). These results suggest that larvae fed on a specific plant species develops or carries symbiotic bacteria possessing a highly efficient enzyme arsenal that allows the insect to rapidly process a specific plant biomass. Thus, we wanted to isolate cellulolytic bacteria from larvae feeding on sugarcane in order to explore their potential as a source of enzymes for efficient sugarcane biomass degradation that could be employed in second generation bioethanol production or other biomass treatments.

## Materials and methods

### Isolation of cellulolytic bacteria from the gut of *Diatraea saccharalis*

Fifth instar larvae of *D. saccharalis* were collected from sugarcane fields at the “Estación Experimental Agroindustrial Obispo Colombres (EEAOC)”, Tucumán, Argentina and surface sterilized with 70% ethanol before guts were aseptically dissected from replicates of ten larvae. Isolated intestines were cut into small pieces, homogenized in an isotonic saline solution before plated on a minimal saline agar medium (Na_2_HPO_4_ 1 g; KH_2_PO_4_ 1 g; MgSO_4_ 0.05 g; NaCl 3 g; CaCl_2_ 0.05 g, (NH_4_)_2_SO_4_ 2 g and agar 20 g in 1 L of H_2_O) containing 0.5% of glucose, finely milled sugarcane bagasse or harvest trash (HT) were added as sole carbon sources. Cultures were incubated for 3 days at 37°C and bacterial colonies able of grow on cellulose as sole carbon source were isolated and cellulose-degrading ability was confirmed by streaking the bacteria on the same minimal saline agar supplemented with 0.5% carboxymethyl cellulose (CMC). After 4 days of incubation at 30°C, the medium was treated with 0.1% (w/v) aqueous Congo red, a specific indicator for cellulose hydrolysis leaving a distained halo where CMC has been degraded (Teather and Wood 1982).

### Identification of bacterial isolates and sequence homology analysis of the *16SrDNA* gene

DNA extraction was performed using the PureLink Genomic DNA Kit (Invitrogen, USA) according to the manufacturer’s instructions. Bacterial universal primers fD1 (5’-CCGAATTCGTCGACAACAGAGTTTGATCCTGGCTCAG-3’) and rP1 (5’-CCCGGGATCCAAGCTTACGGTTACCTTGTTACGACTT-3’) were used to amplify the *16S rDNA* gene from genomic DNA (Weisburg et al. 1991). Polymerase chain reaction (PCR) was performed in a Bio-Rad Mycycler Thermalcycler (Hercules, USA). Each reaction mixture (50 μl) contained 1X reaction buffer, 2 mM MgCl_2_, 0.2uM of each primer, 0.2 mM of dNTPs, 2U of Taq DNA polymerase (Invitrogen, USA) and 40 ng of genomic DNA. PCR cycling parameters were 1 cycle at 95°C (2 min); 35 cycles at 95°C (30 sec), 54°C (30 sec), and 72°C (3 min); and a final cycle at 72°C for 10 min. All amplified DNA products were checked by gel electrophoresis in 1% (w/v) agarose gels stained with GelRed (Biotium, USA). The 1.5Kb amplification products were directly purified from the PCR reaction using the PureLink Quick PCR purification kit (Invitrogen, Germany) according to the manufacturer’s instructions. Purified reactions were sequenced by the Biotechnology Institute Sequencing Service, INTA Castelar using an ABI 3130 Capillary DNA analyzer (Applied Biosystems, USA). All sequences were aligned against the NCBI database and in the RDP II database (Cole et al. 2009) using the BLAST (Basic Local Alignment and Search Tool) algorithm (Altschul et al. 1997). The aligned 16S rDNA sequences were analyzed based on their homologous characteristics and a phylogenetic tree was constructed using the software MAFFT 7 online version (Katoh 2013) using the neighbor-joining option with a bootstrap analysis of 1,000 random replications. A sequence from a *Chlamydia pneumoniae* strain CHT16SR (L06108) was used to root the tree. The resulting *16S rDNA* gene sequences for the isolates *Klebsiella oxytoca* Kd70 TUC-EEAOC, *K. pneumoniae* KdB5 TUC-EEAOC, *K. variicola* KdB1 TUC-EEAOC, *Stenotrophomonas maltophilia* Kd3 TUC-EEAOC, *S. rhizophila* Kd46 TUC-EEAOC, *Bacillus pumilus* Kd109 TUC-EEAOC, *Enterococcus casseliflavus* Kd7 TUC-EEAOC, *Microbacterium hominis* KdL49 TUC-EEAOC, *M. schleiferi* KdL45 TUC-EEAOC were submitted to GenBank under the accession numbers of KM096608, KM096599, KM096598, KM096600, KM096602, KM096605, KM096606, KM096603, KM096601 respectively.

*Klebsiella oxytoca* Kd70 TUC-EEAOC, *Bacillus pumilus* Kd109 TUC-EEAOC, *Stenotrophomonas maltophilia* Kd3 TUC-EEAOC, *Enterococcus casseliflavus* Kd7 TUC-EEAOC have been deposited at the German Collection of Microorganisms and Cell Cultures (DSMZ, http://www.dsmz.de) as accession DSM 27019, DSM 27021, DSM 27017 and DSM 27018 respectively.

### Determination of total cellulase activity

Bacteria were grown overnight in Luria-Bertani Broth (LB) and 20 μl of a bacterial suspension adjusted to an O.D. of 0.2 was thereafter applied as a drop on solid agar plates containing the previously described saline minimal medium supplemented with 0.5% (w/v) finely milled bagasse or sugarcane harvest trash residues. Bacteria were grown for a fortnight at 30°C and plates were thereafter colored with Congo red to determine possible cellulose degradation. The diameter of the clear zone around the bacterial drop is indicative of the magnitude of cellulolytic activity.

### Extracellular CMCase activity assay

Twenty (20) micro liters of a bacterial growth suspension were adjusted to an O.D. of 0.2 and applied, as a drop, on solid minimal medium containing 0.1% glucose and 0.5% (w/v) CMC. Plates were thereafter incubated at 30°C during 10 days before plates were photographed to monitor bacterial growth and subsequently colored with Congo red to visualize extracellular endo-glucanase activity. The enzymatic activity index (EIA) was calculated as the diameter of the clear halo plus the colony diameter/diameter of the colony (Hankin and Anagnostakis 1977; Huang et al. 2012). A bacterium with an EIA above of 2.5 is considered as a producer of cellulolytic enzymes (Anagnostakis and Hankin 1975).

For testing extracellular endo-glucanase activity in liquid medium bacterial cells grown in a rich medium were inoculated in a saline minimal media (described previously) supplemented with 0.2% glucose, 0.2% tryptone and 0.5% (w/v) CMC (adaptation medium) and incubated for 24 h at 37°C. Adapted bacterial cells were inoculated in minimal media with different carbon sources as mentioned above and incubated during 14 days in a shaker incubator at 150 rpm at 30°C. Samples were taken at different time (5, 10, 14 days after inoculation) and centrifuged, first at 5,000 rpm for 15 min and thereafter 20 min at 13,000 rpm. Total protein concentration was quantified by the method of Bradford with the Bio-Rad protein assay kit (Bio-Rad Laboratories, Richmond, CA) using bovine serum albumin as protein standard (Bradford 1976). Two hundred microliter (200 μl) containing 10 μg of total protein were applied in small wells in agar plates containing minimal medium supplemented with 0.5% (w/v) CMC. Plates were thereafter incubated for 10 days at 30°C before overlaid with Congo red. A clear halo around a well indicates extracellular cellulolytic activity.

Endo-cellulolytic enzyme activity was measured using the 3,5-dinitrosalicylic acid (DNS) reagent (Miller 1959). The reaction mixture consisted of 100 μl of 2% (w/v) CMC in 100 mM sodium acetate buffer (pH 5.0), to which 100 μl of the obtained supernatants were added before incubation at 40°C for different time points. To stop the reaction, 200 μl of DNS reagent was added and the reaction mixture was heated at 100°C for 10 min. Next, the mixture was left to cool to room temperature, centrifuged and then the absorbance at 540 nm was measured using a spectrophotometer. One unit of enzymatic activity is defined as the amount of enzyme needed to release 1 μmol of reducing sugars (measured as glucose) per ml/min during the reaction.

### Scanning electron microscopy

The scanning electron microscopy (SEM) was used following the protocol described by Karnovsky (1965). Briefly, 7 days bacterial cultures were harvested from minimal media with 0.5% bagasse or HT and fixed overnight at 4°C in 8% paraformaldehyde, 0.1 M sodium phosphate buffer at pH 7.4 and 25% glutaraldehyde. The suspension was washed in sodium phosphate buffer 3 times for 10 min each and post-fixed in a solution of a 2% osmium tetroxide in sodium phosphate buffer. After fixation samples were stepwise dehydrated with 30%, 50%, 70%, 80%, 90% and 100% concentrations of ethanol, followed by a final treatment using 100% acetone. To preserve the surface structure of the specimen and to avoid damaging due to surface tension during microscopic observation, critical point procedure was used. The sample was then mounted in an aluminum sample holder and covered with a layer of gold and observed under high vacuum on a Zeiss Supra 55 VP (Carl Zeiss, Oberkochen, Germany) scanning electron microscope at the CIME (Centro Integral de Microscopía Electrónica, INSIBIO, Tucumán, Argentina).

## Results

### Isolation of cellulolytic bacteria from the intestine of *Diatraea saccharalis*

**Figure 1:**
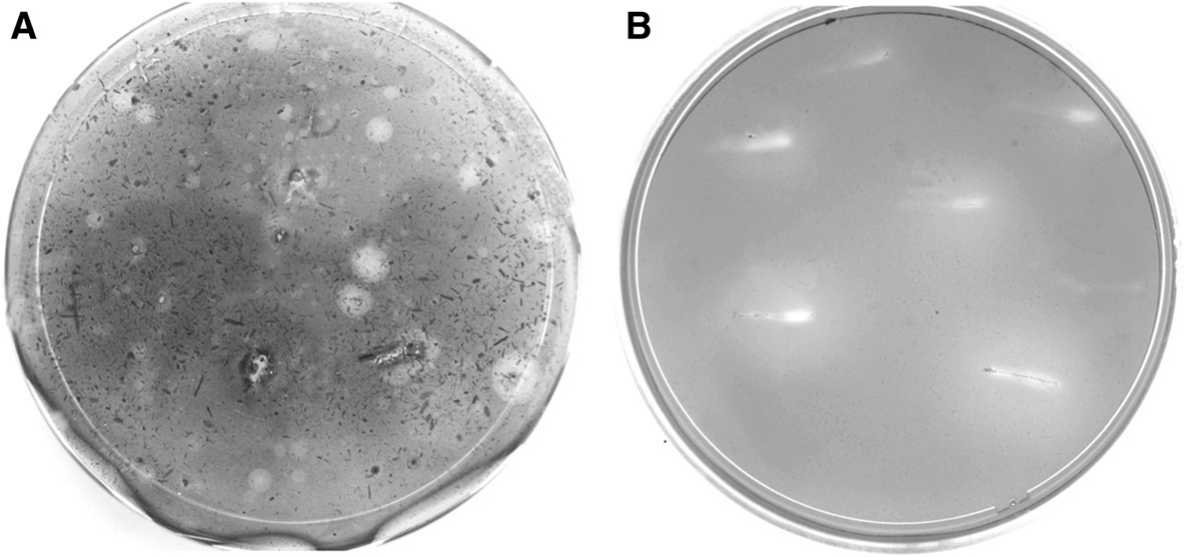
**Screening for sugarcane biomass degrading bacteria. A)** Bacteria from the intestine of *D. saccharalis* were grown for 3 days on Petri dishes containing minimal medium supplemented with 0.5% (w/v) sugarcane bagasse as sole carbon source. After colonies had developed, plates were stained using Congo red dye to indicate cellulolytic activity of the colony. **B)** Bacterial strains showing cellulolytic activity on plates in **A** were purified and grown on Petri dishes containing CMC, which were later coloured with Congo red. The halo around bacterial colonies indicates total cellulolytic activity of the bacterial isolate.

In order to isolate bacterial symbionts of the intestine of sugarcane-fed *D. saccharalis,* fifth instar larvae were collected in sugarcane production fields in the Province of Tucumán in the Northwest of Argentina. Intestines of larvae were immediately dissected and plated on agar plates containing a bacterial minimal growth medium supplemented either with glucose or sugarcane biomass in the form of bagasse or harvest trash (HT), as sole carbon source. From these initial plates 118 bacterial colonies were isolated showing preliminary cellulose degrading activity as deduced from halo formation around the bacterial colony when treated with Congo red (Figure 1A), eighty from glucose plates (1.2 × 10^8^ colony forming units.ml^-1^) and thirty eight from the two lignocellulose substrate plates (2.0 × 10^5^ colony forming units.ml^-1^).

After a round of colony purification all bacterial colonies indicated to possess cellulase activity were re-tested for cellulose degrading capacity using minimal growth medium supplemented with CMC. Thirty eight of the 118 originally isolated bacterial colonies, nineteen from glucose plates and nineteen from sugarcane biomass plates, showed variable zones of clearance surrounding the bacterial colony after incubation with Congo red (Figure 1B) and were selected for further studies.

### Bacterial taxonomy and sequencing analysis of the *16S rDNA* gene

*In vitro* DNA amplification by the polymerase chain reaction (PCR) of the entire *16S rDNA* gene sequence was performed using genomic DNA isolated from the 38 bacterial strains showing cellulolytic activity as a template*.* Amplified DNA fragments of correct size were sequenced and compared to known 16S rDNA sequences in the GenBank DNA database and the Ribosomal Database Project. Sequence alignment allowed us to identify isolates at the genus level and, in some cases at the species level (Table 1).

**Table 1:** **Identification and characterization of bacterial isolates based on 16S rDNA homology analysis and cellulolytic activity**

**Strain**	GenBank/DSMZ accession no.*	**Bacteria**	**Closest relative**	**16S rDNA identity (%)**	**Cellulolytic activity**		
					**Bagasse**	**HT**	**CMC (EIA)**
Kd70 TUC-EEAOC	KM096608/DSM 27019	*Klebsiella oxytoca*	*K. oxytoca* KCTC1686	99.0	+	+	+ (5.0)
KdB5 TUC-EEAOC	KM096599	*Klebsiella. pneumoniae*	*K. pneumoniae* MGH78578	99.0	+	+	+ (5.5)
KdB1 TUC-EEAOC	KM096598/DSM 27017	*Klebsiella variicola*	*K. variicola* At-22	99.7	+	+	+ (5.0)
Kd3 TUC-EEAOC	KM096600	*Stenotrophomonas maltophilia*	*S. maltophilia* R551-3	98.3	-	-	+ (2.5)
Kd46 TUC-EEAOC	KM096602	*Stenotrophomonas rhizophila*	*S. rhizophila* ep-10	99.0	-	-	+ (2.5)
Kd109 TUC-EEAOC	KM096605/DSM 27021	*Bacillus pumilus*	*B. pumilus* SAFR032	99.5	+	+	+ (7.0)
Kd7 TUC-EEAOC	KM096606/DSM 27018	*Enterococcus casseliflavus*	*E. casseliflavus* EC-20	99.0	+ weak	+ weak	+ (2.5)
KdL49 TUC-EEAOC	KM096603	*Microbacterium hominis*	*M. hominis* DSM12509	99.8	-	-	+ (1.5) weak
KdL45 TUC-EEAOC	KM096601	*Microbacterium schleiferi*	*M. schleiferi* DSM20489	97.0	-	-	+ (1.5) weak

Numbers in parenthesis in the column of cellulolytic activity on CMC indicate diameter of halo in centimeters.

**Figure 2:**
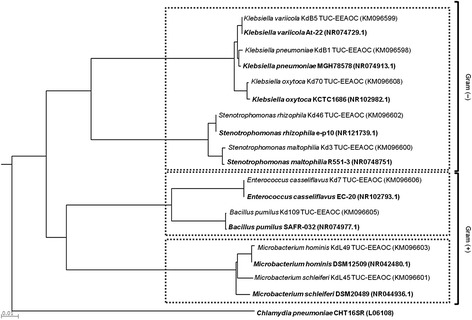
**Neighbour-joining phylogenetic tree using the** ***16S rDNA*** **gene for sequence homology studies.** A phylogenetic relationship from the 38 isolates showing growth and cellulolytic activity on biomass from sugarcane and CMC. Sequences of reference strains obtained from DNA databases are indicated in bold and accession numbers are given in parenthesis. Bacterial isolates outlined in top, middle and bottom groups belong to the phylum Proteobacteria, Firmicutes and Actinobacteria respectively. The scale represents 0.01 substitutions per nucleotide position. Bootstrap values are based on 1,000 replicates.

The corresponding phylogenetic analysis based on the *16S rDNA* gene sequences obtained is presented in Figure 2 where the homology tree shows two main groups, Gram-negative and Gram-positive bacteria. The former group contained isolates belonging to the phylum Proteobacteria, which included species from genera *Stenotrophomonas* (5.2%) and *Klebsiella* (47.4%). The Gram-positive group included bacteria belonging to the phyla Actinobacteria, represented by species of the genus *Microbacterium* (13.2%), and Firmicutes represented by species of the two genera, *Enterococcus* (23.7%) and *Bacillus* (10.5%).

The *16S rDNA* gene sequences of the isolated strains showed nucleotide homology ranging from 97.0 to 99.8% with bacterial strains published in the data base. The Gram-negative bacteria *Klebsiella oxytoca* Kd70 TUC-EEAOC isolate was phylogenetically most closely related to the *K. oxytoca* KCTC1686 strain (99.0% sequence homology). The *Klebsiella pneumoniae* isolate KdB1 TUC-EEAOC showed a 99.0% sequence similarity with *K. pneumoniae* strain MGH78578 whereas *Klebsiella variicola* KdB5 TUC-EEAOC was very closely related to *K. variicola* strain At-22 (99.7% sequence similarity). The *Stenotrophomonas maltophilia* Kd3 TUC-EEAOC isolate shared a 98.3% sequence identity with *S. maltophilia* strain R551-3 and *S. rhizophila* Kd46 TUC-EEAOC showed 99,0% homology with the *S. rhizophila* ep-10 strain.

In the case of the Gram-positive isolates, the Kd109 TUC-EEAOC isolate identified as *Bacillus pumilus* showed a 99.5% of 16S rDNA sequence homology with the *B. pumilus* strain SAFR-032. Isolate KdL49 TUC-EEAOC (*Microbacterium hominis*) is very closely related to strain *M. hominis* DSM-12509 with a 99.8% sequence homology while KdL45 TUC-EEAOC (*M. schleiferi*) is phylogenetically most closely related with the *M. schleiferi* strain DSM-20489 (97.0% of identity). Finally the Kd7 TUC-EEAOC isolate (*Enterococcus casseliflavus*) showed a 99.0% homology with the *E. casseliflavus* strain EC-20.

### Total cellulase activity assay

**Figure 3:**
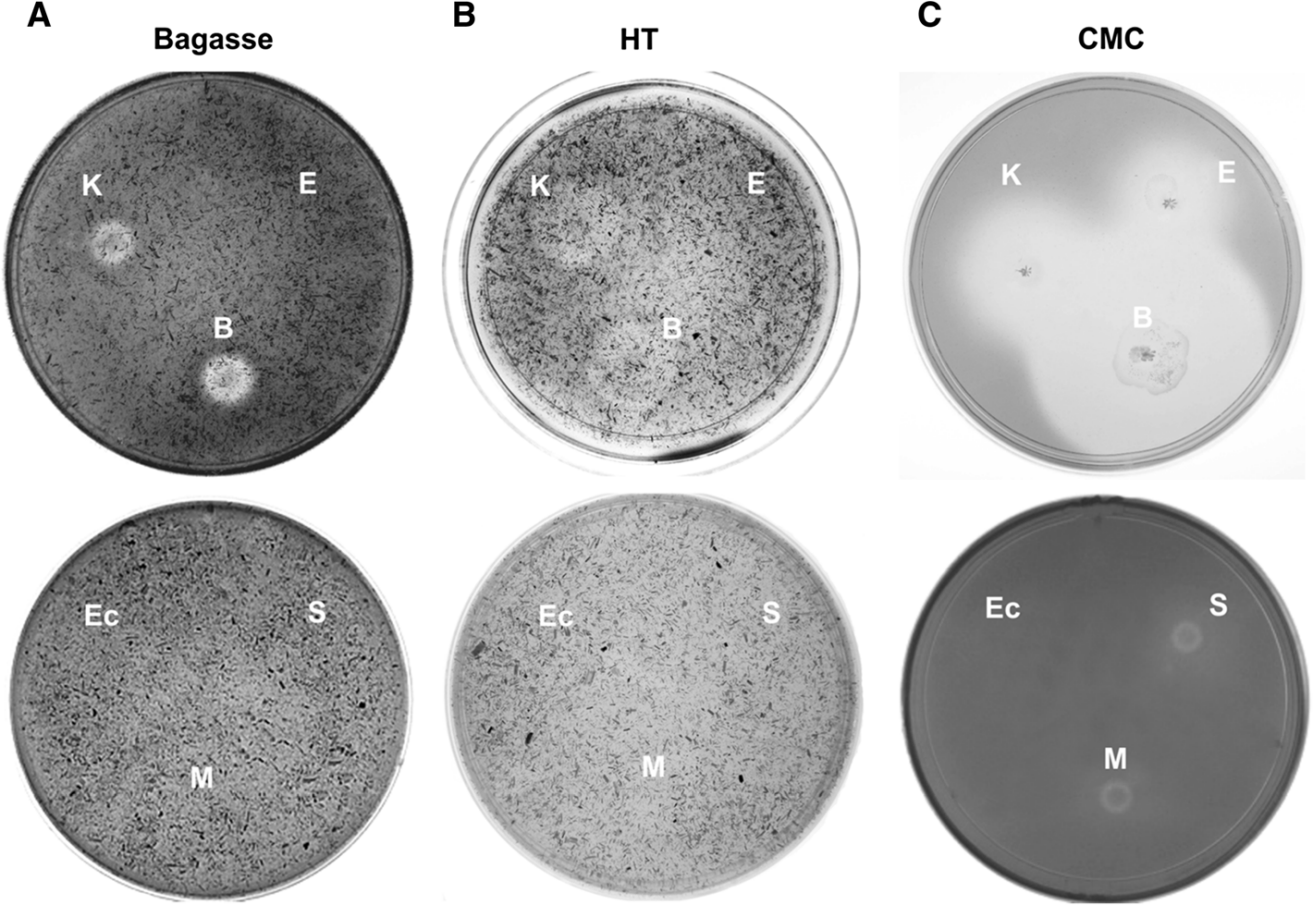
**Qualitative cellulolytic assay of bacterial isolates.** Solid agar plates containing a saline minimal medium supplemented with **(A)** bagasse, **(B)** harvest trash (HT) and **(C)** carboxymethyl cellulose (CMC) as sole carbon sources. Bacteria were let to grow for 15 days at 30°C before treatment with Congo red. Halos around bacterial colonies are indicative of cellulose degradation. Bacteria abbreviations: E = *Enterococcus casseliflavus* strain Kd7 TUC-EEAOC, K = *Klebsiella oxytoca* strain Kd70 TUC-EEAOC and B = *Bacillus pumilus* strain Kd109 TUC-EEAOC, S = *Stenotrophomonas maltophilia* Kd3 TUC-EEAOC, M = *Microbacterium hominis* KdL49 TUC-EEAOC and Ec = *Escherichia coli* DH5α as a negative control.

**Figure 4:**
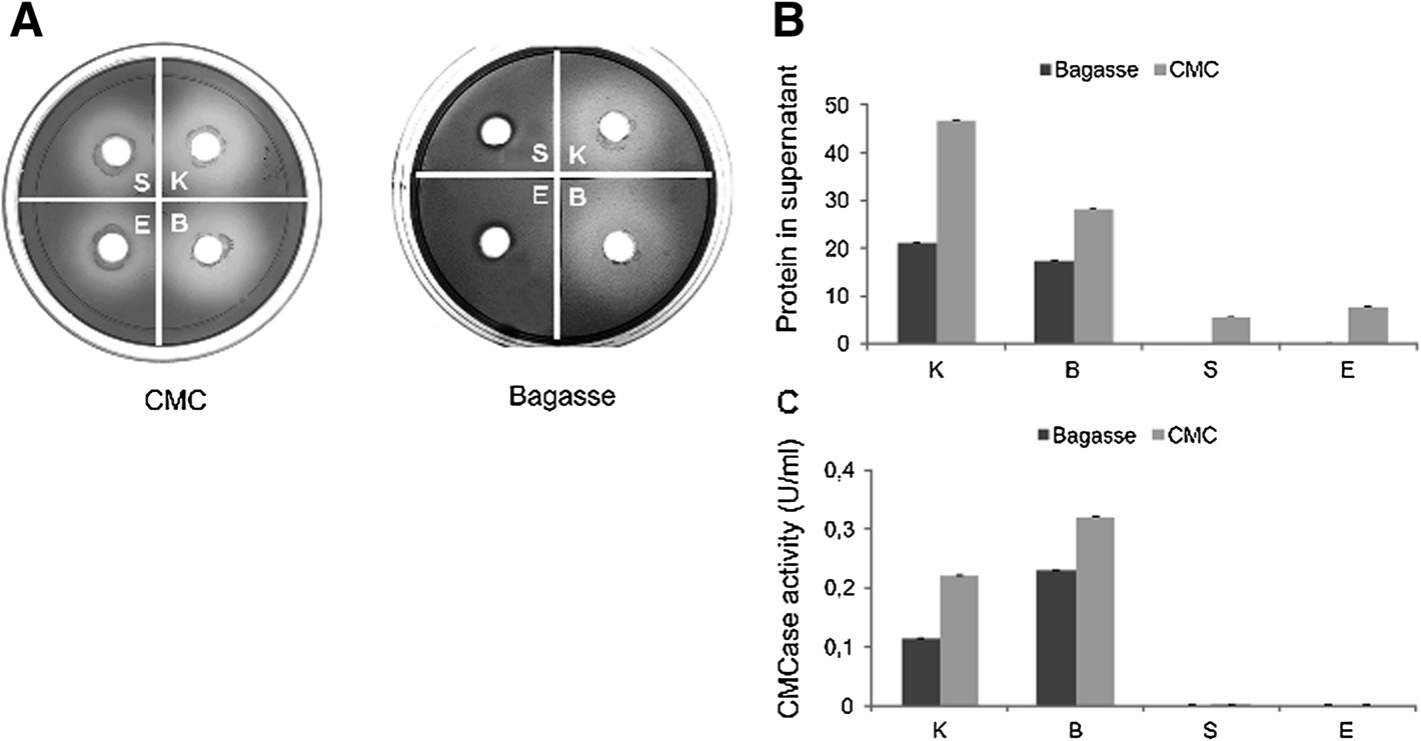
**Measurement of extracellular endo-glucanase activity. A)** Enzyme activity tested through hydrolysis of CMC by supernatants of bacteria grown on CMC and bagasse. Cell and fiber-free supernatants were incubated for 10 days at 30°C before staining with Congo red. Halos around wells indicate extracellular endo-glucanase activity. **B)** Total extracellular protein concentration as measured by the method of Bradford of bacterial cultures incubated in medium containing sugarcane residues or CMC. **C)** CMCase activity in supernatants of bacterial strain selected for high cellulolytic activity grown on CMC and bagasse expressed in units.ml^-1^. Bacterial abbreviations: E = *Enterococcus casseliflavus* Kd7 TUC-EEAOC, K = *Klebsiella oxytoca* Kd70 TUC-EEAOC and B = *Bacillus pumilus* Kd109 TUC-EEAOC, S = *Stenotrophomonas maltophilia* Kd3 TUC-EEAOC.

Endo-glucanases cleave amorphous regions of the cellulose microfibril producing termini of cellulose chains whereas exo-glucanases act on these termini to loosen the crystalline structure of the microfibril. Endo- and exo-glucanase activities are measured using soluble substrates (CMC) and crystalline forms of cellulose (alpha-cellulose or Avicel), respectively. Exo- and endo-glucanase activity was analyzed for the thirty-eight (38) bacterial isolates selected using agar plates containing either bagasse, HT or CMC as carbon sources. After 10 days of growth at 30°C, bacterial plates were stained with Congo red and scored for presence of a clearing zone (halo) around each colony, indicating cellulose degradation. Among the isolates tested, only strains belonging to species of the genera *Klebsiella* and *Bacillus* demonstrated clear total cellulase and endo-glucanase activities (Figure 3A, 3B, 3C and Table 1). Bacteria belonging to species of the genera *Enterococcus* and *Stenotrophomonas* showed marked endo-glucanase activity on CMC plates (Figures 3C and 4A) but despite that they were able to grow on lignocellulose substrates did not show any noticeable clearing zones of hydrolysis on sugarcane biomass substrates (Figures 3A, 3B and 4A). Bacterial strains of species belonging to the genus *Microbacterium* displayed a very low cellulolytic activity when grown on CMC plates and were unable to grow on any of the two lignocellulose substrates from sugarcane tested (Figure 3A and Figure 3B).

As shown in Figure 3C and Table 1 bacterial isolates of *Klebsiella*, *Bacillus, Enterococcus*, *Stenotrophomonas* and *Microbacterium* produced cleared zones of variable size on CMC plates. After 4 days of incubation, the strain of *B. pumilus* generated the largest halo indicating the highest cellulase activity of all the isolated strains tested. The *B. pumilus* strain produced a halo with a 7 cm diameter, while strains belonging to the genera *Klebsiella* produced halos of 5.5, and 5.0 cm in diameter. Finally, strains belonging to species of the genera *Enterococcus*, *Stenotrophomonas* and *Microbacterium* showed much smaller areas of discoloration with diameters ranging from 2.5 to 1.5 cm (Table 1).

To investigate whether isolates of *Klebsiella oxytoca, K. pneumoniae, K. variicola* and *Bacillus pumilus,* which showed the highest cellulolytic activity, were constituted by different genotypes, the repetitive extragenic palindromic elements-PCR fingerprinting technique (rep-PCR) was performed based on cluster analysis combining the ERIC, BOX and REP markers. Genetic diversity studies of the eighteen (18) *Klebsiella* isolates revealed 3 clusters separating the three aforementioned species, but in only one of the species, *Klebsiella oxytoca*, genetic difference among strains were detected where strain Kd-70 TUC-EEAOC was found to be genetically distinct to the other isolates (data not shown). The four isolates of *Bacillus pumilus* were found to consist of two different genotypes which grouped into two clusters each formed by two members (data not shown).

### Extracellular CMCase activity assay

Total endo-glucanase activity produced by the isolated strains was tested in both solid and liquid medium, while extracellular endo-glucanase activity was evaluated using agar plates containing CMC, where 200 μL aliquots of cell and fiber free supernatants obtained from bacterial cultures supplemented with CMC or sugarcane biomass were applied. After incubation at 30°C for 10 days and subsequent staining with Congo red, halo formation around inoculated wells showed extracellular cellulase activity. It is interesting to note that the largest halo diameter, indicating a higher activity (ratio > 5.0), was seen for species of *Klebsiella* and *Bacillus* when cultures were incubated in the presence of either insoluble or soluble cellulosic substrates (Figure 4A), corroborating our results from total cellulase activity measurements (Figure 3A, 3B and 3C).

In contrast, species belonging to the genera *Stenotrophomonas* and *Enterococcus* only showed a moderate extracellular activity when incubated in medium supplemented with CMC and did not show any measureable activity when cultured in insoluble cellulosic substrates (Figure 4A). These results are consistent with the growth and halo profiles obtained on CMC and sugarcane biomass plates in Figure 3A, 3B and 3C. No detectable extracellular cellulase activity was found for species belonging to *Microbacterium* grown in medium containing CMC or sugarcane biomass (data not shown).

When analyzing total protein concentration in the extracellular medium during growth on CMC, highest protein accumulation were found for *Klebsiella oxytoca* Kd70 TUC-EEAOC (46.6 μg/ml) and *Bacillus pumilus* Kd109 TUC-EEAOC (28.3 μg/ml) followed by *Stenotrophomonas maltophilia* Kd3 TUC-EEAOC and *Enterococcus casseliflavus* Kd7 TUC-EEAOC with 5.5 μg/ml and 7.8 μg/ml, respectively (Table 2). When growth was performed in media supplemented with sugarcane bagasse, the same tendency as with CMC was observed. In this case, similar amounts of extracellular protein was secreted by *K. oxytoca* (21.08 μg/ml) and *B. pumilus* (17.47 μg/ml) while in the case of *E. casseliflavus* and *S. maltophilia* very little or no protein was detected in the supernatant (Table 2).

**Table 2:** **Total protein concentrations and CMCase activity determined in the extracellular medium of bacteria isolates**

**Bacteria**	**Total secreted protein (μg.ml-1) ± SD**		**CMCase activity (U.ml** ^**-1**^ **) ± SD**	
	**CMC supernatant**	**Bagasse supernatant**	**CMC supernatant**	**Bagasse supernatant**
*Klebsiella oxytoca* Kd70 TUC-EEAOC	46.65 ± 0.04	21.08 ± 0.04	0.22 ± 0.001	0.13 ± 0.001
*Bacillus pumilus* Kd101 TUC-EEAOC	28.31 ± 0.05	17.47 ± 0.02	0.32 ± 0.002	0.23 ± 0.001
*Stenotrophomonas maltophilia* Kd3 TUC-EEAOC	5.48 ± 0.01	ND	0.0010 ± 0.0002	ND
*Enterococcus casseliflavus* Kd7 TUC-EEAOC	7.79 ± 0.01	ND	ND	ND

ND: No detected under the assay conditions used.

When quantifying CMCase activity, highest enzymatic activities were observed for *B. pumilus* (0.32 U/ml) and *K. oxytoca* (0.22 U/ml), while much lower activities were obtained for strains belonging to *E. casseliflavus* and *S. maltophilia*. The same pattern of enzymatic activity was observed for strains grown on sugarcane biomass with highest values scored for *B. pumilus* (0.23 U/ml) and *K. oxytoca* (0.13 U/ml) whereas *E. casseliflavus* and *S. maltophilia* showed much lower or no detectable CMCase activity (Table 2). Similar values as determined for *K. oxytoca* and *S. maltophilia* were obtained when the other species of *Klebsiella* and *S. rhizophila* were assayed (data not shown).

### Bacterial cells adherence to insoluble cellulosic material

**Figure 5:**
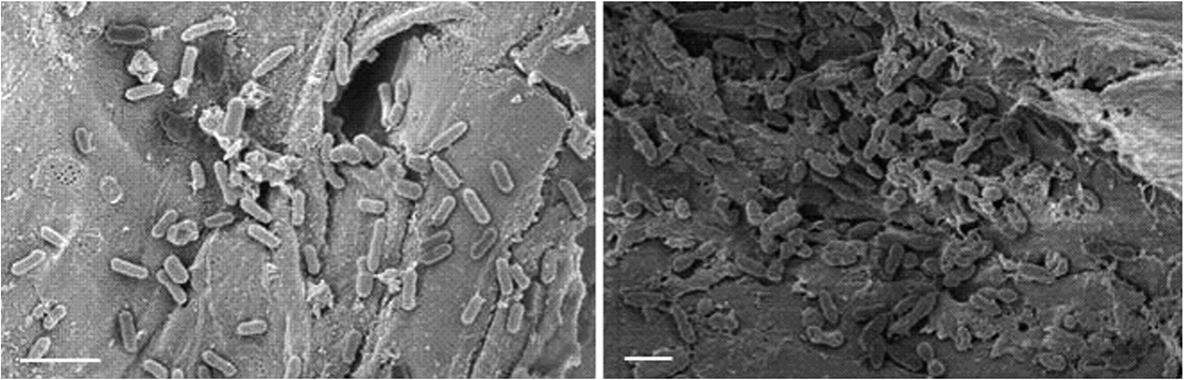
**Bacterial adhesion to sugarcane harvest residues.** Scanning electron microscopy (SEM) images showing bacterial adherence to plant fibers originating from sugarcane HT. Left image, rod-shaped *Klebsiella oxytoca* strain Kd70 TUC-EEAOC. Right image, diplococci of the *Enterococcus casseliflavus* strain Kd7 TUC-EEAOC. White bars shown in the left bottom corners of both figures indicate 2 μm.

An important step for efficient bacterial hydrolysis of cellulose and hemicellulose is the adhesion of the bacteria to the substrate fiber. To verify that bacterial isolates from the intestine of *D. saccharalis* can attach to sugarcane biomass material, scanning electron microscopy studies were performed on bagasse and HT incubated with one Gram-positive (*Enterococcus*) and one Gram-negative bacterium (*Klebsiella*), respectively. As can be seen in Figure 5 both strains attach firmly to both sugarcane bagasse (left) and HT fibers (right). The *Klebsiella oxytoca* strain Kd70 TUC-EEAOC attaches as single rod-shaped bacterial cells while *Enterococcus casseliflavus* strain Kd7 TUC-EEAOC is characteristically found attached in cell-pairs.

## Discussion

Out of a total number of 118 cultivable bacterial isolates obtained from intestines of sugarcane-fed *D. saccharalis*, one third (38) were found to possess cellulolytic activity as determined by degradation of CMC. Homology sequence studies of *16S rDNA* from the 38 samples revealed that all cellulolytic strains belonged to the 3 phyla γ-Proteobacteria, Actinobacteria and Firmicutes, which were represented by species belonging to five different genera: *Klebsiella*, *Stenotrophomonas, Microbacterium, Bacillus* and *Enterococcus*. Members of these genera are wide-spread in nature and all of them have previously been isolated from insect guts (Cardoso et al. 2012; Huang et al. 2012; Shao et al. 2014), shown to possess cellulolytic activity (Teather and Wood 1982; Ohkuma et al. 1995; Charrier et al. 1998; Dillon and Dillon 2004; Prem Anand and Sripathi 2004; Ariffin et al. 2006; Charrier et al. 2006; Mohamed and Huang 2007; Anand et al. 2010; Suen et al. 2010; Okeke and Lu 2011; Ko et al. 2011; Huang et al. 2012) and all except species belonging to the genus *Enterococcus* have been reported as plant endophytes (Zinniel et al. 2002; Asis and Adachi 2004; Dillon and Dillon 2004; Velazquez et al. 2008; Malfanova et al. 2011; Huang et al. 2012; Mingyue et al. 2013; Murugappan et al. 2013; Ren et al. 2013; Chun-Yan et al. 2014; Soni et al. 2014). Taken into account the extensive literature information it seems plausible to conclude that the majority of the cellulolytic bacteria isolated in this study exist in the plant and is colonizing the intestine upon larval feeding of plant tissue. The only genus of bacteria isolated from the *D. saccharalis* gut that has not been reported in plants is *Enterococcus*, which would suggest a colonization of the larval gut from the adult insect via the egg, as has been shown for *Enterococci* in other Lepidoptera larvae (Brinkmann et al. 2008).

It is interesting to notice that many studies of gut microbiota of herbivorous insects are dominated by members of the phyla Actinobacteria, γ-Proteobacteria and Firmicutes, in which the main symbiotic function suggested involve nitrogen fixation, denitrification, carbohydrate degradation, detoxification, and diverse defensive roles against pathogens (Schloss et al. 2006; Pittman et al. 2008; Hernández et al. 2013; Shao et al. 2014). A similar, predominantly nutritive and defense function for the bacteria isolated from the intestine of *D. saccharalis* is highly probable as the immature sugarcane stalks, from where larvae were collected, have a very low nutritional value containing low amounts of nitrogen, proteins, vitamins, sugars and minerals.

To further study and characterize the cellulolytic activity of the CMC-degrading strains isolated from the intestine of *D. saccharalis*, experiments were performed using non-pretreated sugarcane agricultural residues as a substrate in order to reproduce the natural feeding of larvae. These studies revealed that of all the species of the five bacterial genera isolated; only species from *Bacillus* and *Klebsiella* were found to possess a relatively high cellulolytic ability when grown on sugarcane plant biomass indicating that these genera are able to efficiently metabolize both insoluble and soluble cellulose, a prerequisite for complete digestion of lignocellulose biomass. Another interesting observation from the cellulolytic studies of strains belonging to species of these two genera was that sugarcane biomass seemed to induce a higher extracellular enzymatic activity, as deduced by larger halos seen on bagasse and sugarcane HT plates stained with Congo red (cph), compared to CMC and other carbon sources. This observation indicates that there could be a higher specificity and/or efficiency in degrading sugarcane biomass for these bacteria which would support our initial hypothesis. Supporting this observation are studies showing a clear correlation between changes in bacterial gut symbionts with different biomass feed. As an example, larvae of the Lepidoptera *Spodoptera littoralis* fed on artificial feed, lima bean and barley showed a very different composition of bacterial intestinal species in a metagenomic study (Tang et al. 2012). Interestingly, numerous bacteria belonging to the genera *Klebsiella* were found when larvae were fed on barley but were absent in larvae from the artificial feed or only found in very low representation in lima bean-fed insects, which could indicate an important role for species of this genera in larva feeding on grass species, as was seen in our study on sugarcane. However, more studies are needed to elucidate if these bacteria are indeed more efficient in degrading cellulose from a specific biomass by cloning individual cellulase genes and perform *in vitro* studies on enzymatic degradation capacity of individual proteins using different carbohydrate substrates, before any direct conclusion should be drawn. Nevertheless, these results are encouraging and merits further investigation of the efficiency and cellulolytic activity of cellulases and hemicellulases from strains belonging to *Klebsiella* and *Bacillus* species isolated in this study in order to evaluate their efficiency and possible application in sugarcane biomass degradation for 2nd generation bioetanol production or other applications.

The low cellulolytic activity found for bacterial species belonging to species of *Microbacterium*, *Stenotrophomonas* and *Enterococcus* genera suggest primary roles other than carbohydrate degradation in the larval intestine. As mentioned earlier, the importance of *Enterococcus* as an important colonizer of the insect gut is indicated by the early colonization and that *Enterococcus* is categorized as a lactic acid bacterium (LAB), which are known beneficial organisms of the gut microbiota of animals, including insects (Vasquez et al. 2012; Shao et al. 2014). If *Enterococcus* is already existing in the egg of *D. saccharalis* it is probable that this genera plays a very important symbiotic role similar to the one proposed in the metagenomic study of the intestine of the cotton leaf worm, where *Enterococcus* were found to be the most predominant and metabolic active bacteria throughout the entire larval life-cycle indicating the importance of this genus as gut colonizers of larva from members of Lepidoptera (Shao et al. 2014). As one of the likely founder species of the gut microbiota it is interesting to speculate of a possible control role for members of this genus in the establishment of other bacteria in the gut. It is well-known that several members of *Enterococci* attach to the mucus layer of gut epithelium to form a biofilm-like structure (Mohamed and Huang 2007), which could protect and prevent the larval gut from being colonized by pathogenic microbes. As members of *Enterococcus* are also known to produce bacteriocins it is possible that a combined action of biofilm formation and production of antimicrobial compounds helps preventing the entrance and establishing of harmful microorganisms in the larval gut (Ennahar et al. 1998; Ruiz-Rodriguez et al. 2012).

Members of the genus *Microbacterium* have been isolated from air, soil, water, humans and plants (Zinniel et al. 2002; Velazquez et al. 2008; Soni et al. 2014). Strains of *M. testaceum*, isolated from the leaf surface of potato plants were shown to produce N-acylhomoserine lactone (AHL)-degrading enzymes (Morohoshi et al. 2011), indicating a protective role against plant pathogens, which frequently uses quorum sensing strategies when colonizing plant tissue. Another interesting feature reported for *Microbacterium* spp. is the chromium detoxification of plants by reducing the bioavailability of toxic chrome IV from soil irrigated with tannery effluent (Soni et al. 2014). Another member, *M. arborescens*, found in the guts of herbivorous caterpillars produces a powerful iron-binding dps (DNA protection during starvation) protein with peroxidase activity, that has been suggested to prevent the formation of cell-damaging oxygen radicals (Pesek et al. 2011). In addition, the same Dps protein can synthesize and hydrolyze amino acid conjugates (N-acyl-glutamines), which have been shown to trigger the plant defense against insect herbivores (Alborn 1997; Baldwin et al. 2001). This latter characteristic could help the larva to escape plant defense actions by evading the detection signaling.

The genus *Stenotrophomonas*, occur ubiquitously in nature and species like S. *maltophilia* and *S. rhizophila* are often found in the rhizosphere and inside many different plant species where they have been associated with beneficial plant interactions. In contrast to the phylogenetically very closely related genera *Xanthomonas* and *Xylella*, no *Stenotrophomonas* species are known to be phytopathogenic, which make *Stenotrophomonas* spp. excellent candidates for biotechnological applications in agriculture. Furthermore, species of the genus *Stenotrophomonas* play an important ecological role in the nitrogen and sulphur cycles and several species of this genus have a high capacity to metabolize a large range of organic compounds which make them ideal candidates for bio- and phytoremediation. Another important feature of which could be beneficial for the larva is the anti fungal activity described for *S. rhizophila* (Wolf et al. 2002).

In accordance with many recent studies (Watanabe and Tokuda, 2010; Cardoso et al., 2012; Gupta et al., 2012; He et al., 2013; Brune, 2014) our results indicate a very complex interaction between bacteria-insect where the bacterial colonizing of the insect gut seem to help the larva in carbohydrate degradation, nutritional access and uptake, protection against pathogens, evasion of plant defense and possible detoxification of chemical compounds. Our understanding of these interactions are only beginning to unfold and many more studies are needed on individual species together with genetic and chemical manipulations of the insect and bacteria in order to advance our knowledge on the role of the larval gut microbiota in herbivorous insects. It is important not to forget the impact of the plant as most of the bacteria isolated from the larva in this study seem to originate from plant endophytes. If this is the case these bacteria show an interesting and highly adoptive life ecology changing a mutual beneficial interaction with its host to a direct antagonistic role protecting and helping an invasive organism. Further understanding of this tri-trophic plant-insect-bacterial interaction should provide valuable information and discoveries that could be employed in novel and more efficient biotechnological solutions for carbohydrate degradation, pest and disease management and phyto- and bio-remediation for example.

## References

[CR1] Alborn HT (1997). An elicitor of plant volatiles from beet armyworm oral secretion. Science.

[CR2] Altschul SF, Madden TL, Schaffer AA, Zhang J, Zhang Z, Miller W, Lipman DJ (1997). Gapped BLAST and PSI-BLAST: a new generation of protein database search programs. Nucleic Acids Res.

[CR3] Anagnostakis L, Hankin SL (1975). Use of selective media to detect enzyme production by microorganisms in food products. J Milk Food Technol.

[CR4] Anand AA, Vennison SJ, Sankar SG, Prabhu DI, Vasan PT, Raghuraman T, Geoffrey CJ, Vendan SE (2010). Isolation and characterization of bacteria from the gut of *Bombyx mori* that degrade cellulose, xylan, pectin and starch and their impact on digestion. J Insect Sci.

[CR5] Ariffin H, Abdullah N, Kalsom MSU, Shirai Y, Hassan MA (2006). Production and characterization of cellulase by *Bacillus pumilus* EB3. Int J Eng Technol.

[CR6] Asis CA, Adachi K (2004). Isolation of endophytic diazotroph *Pantoea agglomerans* and nondiazotroph *Enterobacter asburiae* from sweetpotato stem in Japan. Lett Appl Microbiol.

[CR7] Baldwin IT, Halitschke R, Kessler A, Schittko U (2001). Merging molecular and ecological approaches in plant–insect interactions. Curr Opin Plant Biol.

[CR8] Bradford MM (1976) A rapid and sensitive method for the quantitation of microgram quantities of protein utilizing the principle of protein-dye binding. Anal Biochem 72:248–254 10.1016/0003-2697(76)90527-3 942051

[CR9] Brinkmann N, Martens R, Tebbe CC (2008). Origin and diversity of metabolically active gut bacteria from laboratory-bred larvae of *Manduca sexta* (Sphingidae, Lepidoptera, Insecta). Appl Environ Microbiol.

[CR10] Brune A (2014). Symbiotic digestion of lignocellulose in termite guts. Nat Rev Microbiol.

[CR11] Canilha L, Chandel AK, Dos Santos Milessi S, Antunes T, Fernandes Antunes AF, Da Costa Freitas LW, Almeida Felipe MG, Silva D, Silverio S (2012) Bioconversion of sugarcane biomass into ethanol: An overview about composition, pretreatment methods, detoxification of hydrolysates, enzymatic saccharification, and ethanol fermentation. J Biomed Biotechnol. doi:10.1155/2012/989572. 10.1155/2012/989572 PMC3516358 23251086

[CR12] Cardoso AM, Cavalcante JJV, Cantao ME, Thompson CE, Flatschart RB, Glogauer AS, Scapin SMN, Sade YB, Beltrao PJMSI, Gerber AL, Martins OB, Garcia ES, de Souza W, Vasconcelos ATR (2012) Metagenomic analysis of the microbiota from the crop of an invasive snail reveals a rich reservoir of novel genes. PLoS One. doi:10.1371/journal.pone.0048505. 10.1371/journal.pone.0048505 PMC3486852 23133637

[CR13] Charrier M, Combet-Blanc Y, Ollivier B (1998). Bacterial flora in the gut of *Helix aspersa* (Gastropoda Pulmonata): evidence for a permanent population with a dominant homolactic intestinal bacterium, *Enterococcus casseliflavus*. Can J Microbiol.

[CR14] Charrier M, Fonty G, Gaillard-Martinie B, Ainouche K, Andant G (2006). Isolation and characterization of cultivable fermentative bacteria from the intestine of two edible snails, *Helix pomatia* and *Cornu aspersum* (Gastropoda: Pulmonata). Biol Res.

[CR15] Cheng H, Wang L (2013). Lignocelluloses Feedstock Biorefinery as Petrorefinery Substitutes, Biomass Now - Sustainable Growth and Use, Dr. Miodrag Darko Matovic (Ed.), ISBN: 978-953-51-1105-4, InTech, doi: 10.5772/51491 (www.intechopen.com/books/biomass-now-sustainable-growth-and-use/lignocelluloses-feedstock-biorefinery-as-petrorefinery-substitutes)

[CR16] Chun-Yan W, Li L, Li-Jing L, Yong-Xiu X, Hu C-J, Yang L-T, Yang-Rui L, An Q (2014). Endophytic nitrogen-fixing *Klebsiella variicola* strain DX120E promotes sugarcane growth. Biol Fertil Soils.

[CR17] Cole JR, Wang Q, Cardenas E, Fish J, Chai B, Farris RJ, Mcgarrell DM, Marsh T, Garrity GM (2009). The ribosomal database project: improved alignments and new tools for rRNA analysis. Nucleic Acids Res.

[CR18] Dillon RJ, Dillon VM (2004). The gut bacteria of insects: nonpathogenic interactions. Annu Rev Entomol.

[CR19] Ennahar S, Aoude-Werner D, Assobhei O, Hasselmann C (1998). Antilisterial activity of enterocin 81, a bacteriocin produced by *Enterococcus faecium* WHE 81 isolated from cheese. J Appl Microbiol.

[CR20] Gupta P, Samant K, Sahu A (2012) Isolation of cellulose-degrading bacteria and determination of their cellulolytic potential. Int J Microbiol. doi:10.1155/2012/578925. 10.1155/2012/578925 PMC3270400 22315612

[CR21] Hankin L, Anagnostakis SL (1977). Solid media containing carboxymethylcellulose to detect CX cellulose activity of micro-organisms. J Gen Microbiol.

[CR22] He S, Ivanova N, Kirton E, Allgaier M, Bergin C, Scheffrahn RH, Kyrpides NC, Warnecke F, S.G.Tringe, Hugenholtz P (2013) Comparative metagenomic and metatranscriptomic analysis of hindgut paunch microbiota in wood- and dung-feeding higher termites. PLoS One. doi:10.1371/journal.pone.0061126. 10.1371/journal.pone.0061126 PMC3625147 23593407

[CR23] Hernández N, Escudero JA, Millán ÁS, González-Zorn B, Lobo JM, Verdú JR, Suárez M (2013) Culturable aerobic and facultative bacteria from the gut of the polyphagic dung beetle *Thorectes lusitanicus* Jeckel. Insect Sci n/a–n/a. doi:10.1111/1744–7917.12094. 10.1111/1744-7917.12094 24339348

[CR24] Huang S, Sheng P, Zhang H (2012). Isolation and identification of cellulolytic bacteria from the gut of *Holotrichia parallela* larvae (Coleoptera: Scarabaeidae). Int J Mol Sci.

[CR25] Karnovsky MJ (1965). A formaldehyde-glutaraldehyde fixative of high osmolarity for use in electron microscopy. J Cell Biol.

[CR26] Katoh S (2013) MAFFT multiple sequence alignment software version 7: improvements in performance and usability. Mol Biol Evol 30:772–780 10.1093/molbev/mst010 PMC3603318 23329690

[CR27] Ko K-C, Han YC, Jong Hyun K, Geun-Joong L, Seung-Goo SJJ (2011). A novel bifunctional endo-/exo-type cellulase from an anaerobic ruminal bacterium. Appl Microbiol Biotechnol.

[CR28] Limayem A, Ricke SC (2012). Lignocellulosic biomass for bioethanol production: current perspectives, potential issues and future prospects. Prog Energy Combust Sci.

[CR29] Malfanova N, Kamilova F, Validov S, Shcherbakov A, Chebotar V, Tikhonovich I, Lugtenberg B (2011). Characterization of *Bacillus subtilis* HC8, a novel plant-beneficial endophytic strain from giant hogweed. Microb Biotechnol.

[CR30] Miller GL (1959). Use of dinitrosalicyclic reagent for determination of reducing sugar. Anal Chem.

[CR31] Mingyue C, Li L, Yanming Z, Li S, Qianli A (2013). Genome sequence of *Klebsiella oxytoca* SA2, an endophytic nitrogen- fixing bacterium isolated from the pioneer grass *Psammochloa villosa*. Genome Announc.

[CR32] Mohamed JA, Huang DB (2007). Biofilm formation by enterococci. J Med Microbiol.

[CR33] Morohoshi T, Wang W-Z, Someya N, Ikeda T (2011). Genome sequence of *Microbacterium testaceum* StLB037, an N-acylhomoserine lactone-degrading bacterium isolated from potato leaves. J Bacteriol.

[CR34] Morone A, Pandey RA (2014). Lignocellulosic biobutanol production: gridlocks and potential remedies. Renew Sustain Energy Rev.

[CR35] Murugappan RM, Benazir Begum S, Raja RR (2013). Symbiotic influence of endophytic *Bacillus pumilus* on growth promotion and probiotic potential of the medicinal plant *Ocimum sanctum*. Symbiosis.

[CR36] Ohkuma M, Noda S, Horikoshi K, Kudo T (1995). Phylogeney of symbiotic methanogens in the gut of the termite *Reticulitermes speratus*. FEMS Microbiol Lett.

[CR37] Okeke BC, Lu J (2011). Characterization of a defined cellulolytic and xylanolytic bacterial consortium for bioprocessing of cellulose and hemicelluloses. Appl Biochem Biotechnol.

[CR38] Pesek J, Buchler R, Albrecht R, Boland W, Zeth K (2011). Structure and mechanism of iron translocation by a Dps protein from *Microbacterium arborescens*. J Biol Chem.

[CR39] Pittman GW, Brumbley SM, Allsopp PG, O’Neill SL (2008). Assessment of gut bacteria for a paratransgenic approach to control *Dermolepida albohirtum* larvae. Appl Environ Microbiol.

[CR40] Prem Anand A, Sripathi K (2004). Digestion of cellulose and xylan by symbiotic bacteria in the intestine of the Indian flying fox (*Pteropus giganteus*). Comp Biochem Physiol A Mol Integr Physiol.

[CR41] Ren JH, Li H, Wang YF, Ye JR, Yan AQ, Wu XQ (2013). Biocontrol potential of an endophytic *Bacillus pumilus* JK-SX001 against poplar canker. Biol Control.

[CR42] Ruiz-Rodriguez M, Valdivia E, Martin-Vivaldi M, Martin-Platero AM, Martinez-Bueno M, Mendez M, Peralta-Sanchez JM, Soler JJ (2012) Antimicrobial activity and genetic profile of enteroccoci isolated from hoopoes uropygial gland. PLoS One. doi:10.1371/journal.pone.0041843. 10.1371/journal.pone.0041843 PMC3404078 22911858

[CR43] Schloss PD, Delalibera I, Handelsman J, Raffa KF (2006). Bacteria associated with the guts of two wood-boring beetles: *Anoplophora glabripennis* and *Saperda vestita* (Cerambycidae). Environ Entomol.

[CR44] Shao Y, Arias-Cordero E, Guo H, Bartram S, Boland W (2014). In vivo Pyro-SIP assessing active gut microbiota of the cotton leafworm, *Spodoptera littoralis*. PLoS One.

[CR45] Shi W, Ding SY, Yuan JS (2011). Comparison of insect gut cellulase and xylanase activity across different insect species with distinct food sources. Bioenergy Res.

[CR46] Sierra R, Holtzapple MT, Granda CB (2011). Long-term lime pretreatment of poplar wood. AIChE J.

[CR47] Singh A, Hayashi K (1995). Microbial cellulases: protein architecture, molecular properties, and biosynthesis. Adv Appl Microbiol.

[CR48] Soni SK, Singh R, Awasthi A, Kalra A (2014). A Cr(VI)-reducing *Microbacterium* sp. strain SUCR140 enhances growth and yield of *Zea mays* in Cr(VI) amended soil through reduced chromium toxicity and improves colonization of arbuscular mycorrhizal fungi. Environ Sci Pollut Res Int.

[CR49] Suen G, Scott JJ, Aylward FO, Adams SM, Tringe SG, Pinto-Tomas AA, Foster CE, Pauly M, Weimer PJ, Barry KW, Goodwin LA, Bouffard P, Li L, Osterberger J, Harkins TT, Slater SC, Donohue TJ, Currie CR (2010) An insect herbivore microbiome with high plant biomass-degrading capacity. PLoS Genet. doi:10.1371/journal.pgen.1001129. 10.1371/journal.pgen.1001129 PMC2944797 20885794

[CR50] Sun J-Z, Scharf ME (2010). Exploring and integrating cellulolytic systems of insects to advance biofuel technology. Insect Sci.

[CR51] Taherzadeh MJ, Karimi K (2007). Enzyme-based hydrolysis processes for ethanol from lignocellulosic materials: a review. BioResources.

[CR52] Tang X, Freitak D, Vogel H, Ping L, Shao Y, Cordero EA, Andersen G, Westermann M, Heckel DG, Boland W (2012) Complexity and variability of gut commensal microbiota in polyphagous lepidopteran larvae. PLoS One. doi:10.1371/journal.pone.0036978. 10.1371/journal.pone.0036978 PMC3398904 22815679

[CR53] Teather RM, Wood PJ (1982). Use of Congo red-polysaccharide interactions in enumeration and characterization of cellulolytic bacteria from the bovine rumen. Appl Environ Microbiol.

[CR54] Teeri TT (1997). Crystalline cellulose degradation: new insight into the function of cellobiohydrolases. Trends Biotechnol.

[CR55] Vasquez A, Forsgren E, Fries I, Paxton RJ, Flaberg E, Szekely L, Olofsson TC (2012) Symbionts as major modulators of insect health: Lactic acid bacteria and honeybees. PLoS One. doi:10.1371/journal.pone.0033188. 10.1371/journal.pone.0033188 PMC3299755 22427985

[CR56] Velazquez E, Rojas M, Lorite MJ, Rivas R, Zurdo-Pineiro JL, Heydrich M, Bedmar EJ (2008). Genetic diversity of endophytic bacteria which could be find in the apoplastic sap of the medullary parenchym of the stem of healthy sugarcane plants. J Basic Microbiol.

[CR57] Watanabe H, Tokuda G (2010). Cellulolytic systems in insects. Annu Rev Entomol.

[CR58] Weisburg WG, Barns SM, Pelletier D, Lane DJ (1991). 16S ribosomal DNA amplification for phylogenetic study. J Bacteriol.

[CR59] Wolf A, Fritze A, Hagemann M, Berg G (2002). *Stenotrophomonas rhizophila* sp. nov., a novel plant-associated bacterium with antifungal properties. Int J Syst Evol Microbiol.

[CR60] Zinniel DK, Lambrecht P, Harris NB, Feng Z, Kuczmarski D, Higley P, Ishimaru CA, Arunakumari A, Barletta RG, Vidaver AK (2002). Isolation and characterization of endophytic colonizing bacteria from agronomic crops and prairie plants. Appl Environ Microbiol.

